# Complex Physiological Response of Norway Spruce to Atmospheric Pollution – Decreased Carbon Isotope Discrimination and Unchanged Tree Biomass Increment

**DOI:** 10.3389/fpls.2016.00805

**Published:** 2016-06-09

**Authors:** Vojtěch Čada, Hana Šantrůčková, Jiří Šantrůček, Lenka Kubištová, Meelis Seedre, Miroslav Svoboda

**Affiliations:** ^1^Department of Forest Ecology, Faculty of Forestry and Wood Sciences, Czech University of Life Sciences PraguePrague, Czech Republic; ^2^Faculty of Science, University of South Bohemia in České BudějoviceČeské Budějovice, Czech Republic

**Keywords:** climate change, carbon dynamics, growth trends, soil acidification, spruce decline, tree-ring analysis, tree stress

## Abstract

Atmospheric pollution critically affects forest ecosystems around the world by directly impacting the assimilation apparatus of trees and indirectly by altering soil conditions, which subsequently also leads to changes in carbon cycling. To evaluate the extent of the physiological effect of moderate level sulfate and reactive nitrogen acidic deposition, we performed a retrospective dendrochronological analysis of several physiological parameters derived from periodic measurements of carbon stable isotope composition (^13^C discrimination, intercellular CO_2_ concentration and intrinsic water use efficiency) and annual diameter increments (tree biomass increment, its inter-annual variability and correlation with temperature, cloud cover, precipitation and Palmer drought severity index). The analysis was performed in two mountain Norway spruce (*Picea abies*) stands of the Bohemian Forest (Czech Republic, central Europe), where moderate levels of pollution peaked in the 1970s and 1980s and no evident impact on tree growth or link to mortality has been reported. The significant influence of pollution on trees was expressed most sensitively by a 1.88‰ reduction of carbon isotope discrimination (Δ^13^C). The effects of atmospheric pollution interacted with increasing atmospheric CO_2_ concentration and temperature. As a result, we observed no change in intercellular CO_2_ concentrations (Ci), an abrupt increase in water use efficiency (iWUE) and no change in biomass increment, which could also partly result from changes in carbon partitioning (e.g., from below- to above-ground). The biomass increment was significantly related to Δ^13^C on an individual tree level, but the relationship was lost during the pollution period. We suggest that this was caused by a shift from the dominant influence of the photosynthetic rate to stomatal conductance on Δ^13^C during the pollution period. Using biomass increment-climate correlation analyses, we did not identify any clear pollution-related change in water stress or photosynthetic limitation (since biomass increment did not become more sensitive to drought/precipitation or temperature/cloud cover, respectively). Therefore, we conclude that the direct effect of moderate pollution on stomatal conductance was likely the main driver of the observed physiological changes. This mechanism probably caused weakening of the spruce trees and increased sensitivity to other stressors.

## Introduction

Atmospheric pollution and particularly acid sulfate and reactive nitrogen depositions influence ecosystem functioning and services such as carbon sequestration, water purification and nutrient cycling around the world (e.g., Oulehle et al., 2011). While sulfur dioxide (SO_2_) emissions have been successfully regulated since the end of the 20th century in Europe and North America, they are increasing in other parts of the world (Klimont et al., 2013). Emissions of nitrogen oxides (NO_x_) and ammonia (NH_3_) have also decreased since the end of the 20th century in Europe, but remain at relatively high levels compared to the pre-industrial period (Hůnová, 2014).

Low levels of sulfur and nitrogen deposition have a fertilizing effect on plants (Meng et al., 1994; Högberg et al., 2006), while increased levels of deposition can acidify soils causing soil nutrient depletion and toxic aluminium (Al^3+^) mobilization that can weaken the tree root system and cause nutrient deficiency or water stress (Jentschke et al., 2001; Hruška et al., 2012). High levels of deposition directly damage foliage by entering the intercellular space through the stomata and decrease photosynthetic rate, stomatal conductance (Meng et al., 1994) and alter plant water use efficiency (Thomas et al., 2013). Evergreen conifers covering large areas of temperate forests are especially sensitive to atmospheric pollution (Greaver et al., 2012) because the relatively larger surface area of their leaves (also retained during the winter season) effectively captures the deposition of pollutants from the atmosphere (Kopáček and Hruška, 2010). In this study, we examine the extent of the pollution physiological effects on conifers subjected to moderate pollution load, which is not satisfactorily understood at present. This is achieved by characterizing tree physiology using variables derived from plant carbon stable isotope composition and tree biomass increment.

Forest biomass production also plays a fundamental role in the global carbon cycle and forests represent a major terrestrial storage and sink of atmospheric CO_2_ (Pregitzer and Euskirchen, 2004). Therefore, mechanistic understanding of complex environmental effects (including pollution) on forest biomass production is needed (Pregitzer and Euskirchen, 2004). For example, some studies predict an increase in biomass production due to a possible fertilizing effect of increased atmospheric CO_2_ (Peñuelas et al., 2011; Saurer et al., 2014). However, if other limiting factors such as atmospheric pollution or temperatures overwhelm the CO_2_ effect, very complex and variable responses could be observed (Rydval and Wilson, 2012; Treml et al., 2012; Thomas et al., 2013). This study improves understanding of Norway spruce biomass production by examining its temporal trends and inter-annual variations in relation to air pollution and carbon isotope discrimination.

Environmental changes such as air pollution and climate change can alter the importance of different factors limiting tree physiological processes. Dendrochronological studies have shown for example that temperature limited trees can become insensitive to temperature as a result of pollution load (Elling et al., 2009) or can become sensitive to drought in older ages, under higher competition pressure or in warmer climate (Primicia et al., 2015). More importantly, if a change in the correlation is detected during the peak in air pollution, it may indicate changes in water stress (correlation with precipitation or drought index) and/or photosynthesis limitation by climate (correlation with temperature or cloud cover). Increased water stress could be a consequence of pollution related root weakening and decreased temperature/cloud cover response could be a consequence of pollution related photosynthesis limitation by nutrient deficiency or direct foliage damage.

**FIGURE 1:**
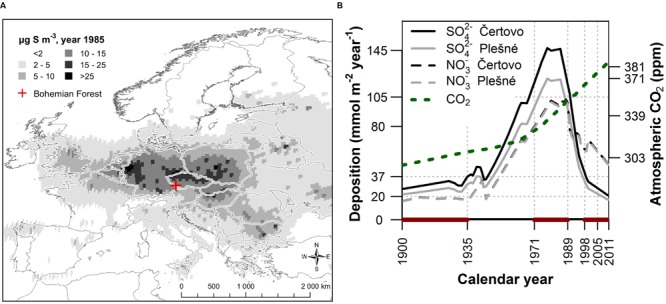
**(A)** European atmospheric SO_2_ concentrations in 1985 (http://www.emep.int) and the location of the moderately polluted study area in the Bohemian Forest situated outside the most severely polluted “Black Triangle” spanning the northern Czech Republic, eastern Germany and southern Poland. **(B)** Temporal trends of SO_4_^2-^ and NO_3_^-^ deposition (Kopáček and Hruška, 2010) together with the increasing trend of atmospheric CO_2_ concentration (McCarroll and Loader, 2004), highlighting selected periods before (1900 – 1935), during (1971 – 1989) and after (1998 – 2005 and 2006 – 2011) the peak in air pollution.

In central Europe atmospheric pollution peaked in the 1970s and 1980s with highest levels in the region called the “Black Triangle,” where widespread mortality and reduction in tree growth, particularly in Norway spruce stands, occurred (Kandler and Innes, 1995; Sander et al., 1995; Rydval and Wilson, 2012). Although the direct link between air pollution and forest decline is evident in the “Black Triangle,” there is lack of evidence about the pollution effect from moderately polluted areas beyond the “Black Triangle” (see **Figure 1**; Kandler and Innes, 1995). The unique study from a moderately polluted area by Šantrůčková et al. (2007) indicated increased stress of Norway spruce trees exposed to moderate pollution load by the analysis of carbon isotope discrimination. Previous studies also often focused on a single physiological parameter (e.g., Šantrůčková et al., 2007; Rydval and Wilson, 2012). In this work we will expand on previous studies with the analysis of the complex response of spruce trees to air pollution as indicated by several physiological variables.

The primary goal of this study is to investigate the extent to which moderate air pollution affected the physiology of mountain Norway spruce in the Bohemian Forest in central Europe. The physiological response is represented using variables obtained from tree-ring carbon isotope composition [^13^C discrimination, intercellular CO_2_ concentration (Ci) and intrinsic water use efficiency (iWUE)] and tree-ring increment (biomass increment, its inter-annual variability and correlation with climate). We specifically aim to address the following questions:

(1) Did the temporal trend of selected spruce physiological parameters follow the temporal trend of air pollution?
(2) What is the relationship between biomass increment and carbon isotope discrimination within tree individuals?
(3) Did biomass increment-climate (temperature, cloud cover, precipitation and Palmer drought severity index) correlations changed during the peak in air pollution?

## Materials and Methods

### Study Area

The study was conducted in the mountain range called the Bohemian Forest in central Europe located along the borders of the Czech Republic, Germany (Bavaria), and Austria (**Figure 1A**). This area was affected by significant pollution load, which peaked in the 1970s and 1980s, although the pollution levels were relatively lower compared to other areas of Central Europe. Sulfur emissions reached values of 10–15 μg S m^-3^ in comparison to more than 25 μg S m^-3^ in the most severely polluted areas such as the Ore Mts. (**Figure 1A**)^1^. In the 1970s and 1980s, deposition of SO_4_^2-^ and NO_3_^-^ was around 125 and 95 mmol m^-2^ year^-1^, respectively, based on model results, which is more than 10 and 25 times higher compared to pre-industrial conditions (**Figure 1B**; Kopáček and Hruška, 2010). To account for this temporal pattern, we divided the data into four periods; before (1900 – 1935), during (1971 – 1989) and after (1998 – 2005 and 2006 – 2011) the period of maximum air pollution. The period after the peak in air pollution was split into two periods to analyze the pattern of forest recovery. By comparing the various tree physiological parameters between these periods we characterize their change over time (hereafter referred to as temporal trend) and association with air pollution.

Our study focuses on natural mountain Norway spruce [*Picea abies* (L.) Karst.] forest, which are present at high elevations of the mountain range. Norway spruce dominates the tree layer (>90%) with minor components of *Sorbus aucuparia* L., *Abies alba* Mill., and *Fagus sylvatica* L.. The understorey is mostly dominated by *Calamagrostis villosa* (Chaix) J. F. Gmel., *Vaccinium myrtillus* L. and *Athyrium distentifolium* Tausch ex Opiz (Svoboda et al., 2006). A large part of the forest comprises of natural old-growth forest, which developed under a natural disturbance regime and which was recently affected by extensive disturbances (Čada et al., 2016).

We selected two glacial-lake catchments 65 km apart; Čertovo (49°10′N, 13°12′E, 1027 – 1343 m.a.s.l.) in the NW and Plešné (48°47′N, 13°51′E, 1089 – 1378 m.a.s.l.) in the SE part of the mountain range. These lakes and catchments are subjected to long-term ecosystem monitoring and research (e.g., Kopáček and Vrba, 2006; Vrba et al., 2014; Kaňa et al., 2015). The bedrock of the Čertovo and Plešné catchments is characterized by poor mica schist and richer granite, respectively, (Cháb et al., 2007). Soils in the area mostly include acidic leptosols, podsols, and dystric cambisols with a higher level of base saturation in the Plešné catchment (Šantrůčková et al., 2007). The climate is cold and humid with mean annual temperatures of around 4°C (Turek et al., 2014), and mean annual precipitation above 1200 mm year^-1^ (CRU TS3.10; Harris et al., 2014).

### Data Collection

Samples for the stable carbon isotopic analyses of wood were collected along a slope transect in both catchments. We extracted increment cores at breast height (1.3 m) from 25 and 6 dominant and healthy (without signs of injury or defoliation) trees in the Čertovo and Plešné catchment, respectively. Samples were analyzed separately for each individual tree. Cores were sectioned into 1–5-year segments, dried and homogenized to a fine powder in a ball mill (MM200 Retsch, Haan, Germany). Carbon isotope composition was determined using an elemental analyzer (EA1110, ThermoQuest, Italy) linked to DeltaXLplus (ThermoFinnigan, Bremen, Germany) for each 1–5-year sample of bulk wood, because the bulk wood provides unbiased estimates in comparison to cellulose composition (Harlow et al., 2006). The ^13^C/^12^C isotopic ratio was calculated relative to the Vienna-Pee Dee Belemnite (VPDB) standard to obtain δ^13^C (McCarroll and Loader, 2004). Trees sampled for isotopic analysis recruited to breast height between 1728 and 1878 (median 1828). We therefore believe that the isotopic composition in the studied period after 1900 was not affected by a juvenile effect.

Samples for biomass increment analysis were collected using a regular grid set across the forest stand. We utilized part of the published datasets of Seedre et al. (2015) and Svoboda et al. (2012) for Čertovo and Plešné catchment, respectively. The regular grid was set across the Čertovo catchment, while the Plešné catchment samples come from a 20 ha plot 2 km from the lake. Increment cores were taken at breast height from 105 canopy trees in both localities. Cores were air-dried, glued to wooden mounts and cut with a razor blade. Ring widths were measured to the nearest 0.01 mm using a sliding table LINTAB and TsapWin software (RINNTECH, Heidelberg, Germany)^2^. Each tree-ring series was cross-dated visually and also using statistical tests implemented in Past4 (Knibbe, 2007; SCIEM, Vienna, Austria). We only used samples which could be reliably cross-dated. All series began before 1900 and median recruitment to breast height was 1859 for the Čertovo and 1814 for the Plešné catchment. Mean sensitivity was 0.21 and 0.20 and mean first order autocorrelation reached 0.86 and 0.88 at the Plešné and Čertovo catchment, respectively. Distance to the pith for cores that did not intersect the pith was estimated using the curvature of the innermost rings and concentric circles printed on transparent foil. Tree age was then estimated by subtracting from the innermost dated ring the estimated distance to the pith divided by the width of the five innermost rings along with 10 additional years to roughly account for the time that a spruce takes to grow from the stump height (30 cm) to the coring height (130 cm).

### Physiological Parameters

Plants obtain their carbon from the atmosphere, yet their ^13^C/^12^C isotopic ratio is reduced relative to CO_2_ in air. This process, called carbon isotope discrimination (against heavier ^13^C isotopes), yields variable isotope ratios depending on the specific plant response to the environment (e.g., irradiance, drought, temperature). CO_2_ diffusion through stomata and photosynthetic carbon fixation are the main processes involved in carbon isotope discrimination. The lighter ^12^C isotope diffuses more easily than the heavier ^13^C and is preferred during fixation by the carboxylation enzyme (Farquhar et al., 1982; McCarroll and Loader, 2004). The leaf internal CO_2_ concentration is also intimately related to ^13^C discrimination because the relative proportion between the CO_2_ influx through stomata and photosynthetic rate control both variables. For example, if the photosynthetic rate is higher than stomatal CO_2_ influx, the internal CO_2_ concentration decreases, and ^13^C discrimination decreases likewise because internal proportion of ^13^C increases (Farquhar et al., 1982; McCarroll and Loader, 2004). By opening their stomata, plants not only obtain CO_2_ for photosynthesis but also loose water vapor. Therefore, plant carbon isotope composition is proportional to water-use efficiency (Farquhar et al., 1982; McCarroll and Loader, 2004).

The carbon isotope ratio of sugars synthetized during photosynthesis is imprinted in plant tissues produced during a given season and we reconstructed the annually integrated spruce physiological parameters (i.e., discrimination against ^13^C, Ci and iWUE) from the carbon isotope ratio of wood. We reconstructed carbon isotope composition of tree foliage using the relationship (δ^13^C_foliage_ = 1.0523 ∗ δ^13^C_wood_ - 0.205) presented in Gebauer and Schulze (1991) and expressed in terms of discrimination against ^13^C in the atmosphere (Δ^13^C) while accounting for the anthropogenic increase in ^12^C atmospheric concentrations (McCarroll and Loader, 2004):

(1) $$\Delta^{13}C\;\left(‰\right)\;=\;\frac{\left(\;\delta^{13}C_{\mathrm{air}}-\;\delta^{13}C_{\mathrm{foliage}}\right)}{\left(1\;-\;\delta^{13}C_{\mathrm{foliage}})/1000\right)}$$

where δ^13^C_air_ and δ^13^C_foliage_ is the relative isotopic composition of the atmosphere and foliage, respectively, for each 5-year segent. Values of δ^13^C_air_ were interpolated from the Law Dome ice cores (McCarroll and Loader, 2004). To reveal the amount of CO_2_ supply for photosynthesis in the conditions of increasing atmospheric CO_2_ concentrations, we calculated the seasonally integrated Ci according to Farquhar et al. (1982):

(2) $$\mathrm{Ci}\;(\mu \mathrm{mol}\;{\mathrm{CO}}_{2}\;{\mathrm{mol}}^{-1}\mathrm{air})\;=\;\frac{\mathrm{Ca}\;*\;\left(\Delta^{13}C\;-\;a\right)}{\left(b\;-\;a\right)}$$

where Ca is the atmospheric CO_2_ concentration in the relevant years, ‘a’ and ‘b’ are constants representing the fractionation during diffusion of CO_2_ through the stomata (4.4‰) and during carboxylation (27‰); values of Ca were obtained from the Law Dome ice cores (McCarroll and Loader, 2004). iWUE reflects the proportion of carbon assimilated in photosynthesis (A) in relation to the potential water loss through stomata (stomatal conductance to water vapor, g_w_), i.e., iWUE = A/g_w_. iWUE was calculated according to McCarroll and Loader (2004):

(3) $$\mathrm{iWUE}\;(\mu \mathrm{mol}\;{\mathrm{CO}}_{2}\;{\mathrm{mol}}^{-1}H_{2}O)=\;\left(\mathrm{Ca}\;-\;\mathrm{Ci}\right)\;*\;0.625$$

where the constant 0.625 reflects the ratio of CO_2_ and H_2_O diffusivity based on the assumption that A = (Ca – Ci) ∗ g_c_, where g_c_ is the stomatal conductance to CO_2_.

Biomass increments were calculated for each calendar year between 1900 and 2006 (2007) for the Čertovo (Plešné) catchment based on ring width series, which were converted to diameters of each year proceeding from pith to bark (including the estimated distance to the pith). The diameters were multiplied by a constant of 1.096 to account for bark thickness and water loss. This value was obtained by comparing the diameters in the final year with actual diameter measurements (**Table 1**). Total tree biomass (needles + branches + dry branches + stem + roots) was calculated using the obtained diameters, ages and modeled heights and crown lengths (**Table 1**) based on best available models developed by Wirth et al. (2004). We used two separate models for height/diameter relationships for the Čertovo and Plešné catchments using data from Svoboda and Pouska (2008) and Seedre et al. (2015), respectively, and one model for the crown length/diameter relationship using data published in Seedre et al. (2015) from the Čertovo catchment, which was considered sufficient because the trends between the two catchments were expected to be similar and because crown length has a relatively minor influence on total biomass. Finally, the biomass increment was obtained by subtracting the biomass of the preceding year from that of the current year. Mean correlation between individual tree biomass increment series for 1900–2007 was 0.20 for the Čertovo and 0.23 for the Plešné catchments. The inter-annual variability of biomass increment was evaluated using a standard dendrochronological metric called sensitivity (Fritts, 1976), which was calculated as:

**Table 1:** Formulas and model descriptions used for biomass calculation.

Formula	*N*	*r*	*p*	Source
**Age** (*years*) = calendar year – (first ring – pith distance/five innermost ring-widths mean – 11)	–	–	–	–
**Diameter** (*mm*) = 1.096^∗^(2^∗^Σring-widths + 2^∗^pith-distance)	129	0.90	<0.0001	Svoboda and Pouska, 2008, this study
**Height** (Čertovo, *m*) = 1.3 + 0.08661^∗^diameter - 6.975E-5^∗^diameter^2^ + 2.137E-8^∗^diameter^3^	1132	0.88	<0.0001	Seedre et al., 2015
**Height** (Plešné, *m*) = 1.3 + 0.05763^∗^diameter - 2.596E-5^∗^diameter^2^	736	0.98	<0.0001	Svoboda and Pouska, 2008
**Crown length** (*m*) = 0.0894^∗^diameter^0.8022^	1128	0.75	<0.0001	Seedre et al., 2015
ln[**biomass-needles** (*kg*)] = 1.07410095^∗^[-1.18863 + 3.33792^∗^LN(diameter/10) - 0.24482^∗^LN(diameter/10)^2^ - 3.31885^∗^LN(height) + 0.49368^∗^LN(height)^2^ - 0.13463^∗^LN(age) + 0.85797^∗^LN(crown length)]				Wirth et al., 2004
ln[**biomass-branches** (*kg*)] = 1.20383259^∗^[0.61063 + 2.40589^∗^LN(diameter/10) - 3.65994^∗^LN(height) + 0.4398^∗^LN(height)^2^ + 0.91027^∗^LN(crown length)]				Wirth et al., 2004
ln[**biomass-dry branches** (*kg*)] = 1.22896995^∗^[-3.09062 + 2.04823^∗^LN(diameter/10) - 1.286761^∗^LN(height) + 0.62836^∗^LN(age)]				Wirth et al., 2004
ln[**biomass-stem** (*kg*)] = 1.0097234^∗^[-2.83958 + 2.55203^∗^LN(diameter/10) - 0.14991^∗^LN(diameter/10)^2^ - 0.19172^∗^LN(height) + 0.25739^∗^LN(height)^2^ - 0.08278^∗^LN(age)]				Wirth et al., 2004
ln[**biomass-roots** (*kg*)] = 1.0526828^∗^[-8.15491 + 4.08262^∗^LN(diameter/10) - 0.28378^∗^LN(diameter/10)^2^ + 0.34963^∗^LN(age) + 0.2452^∗^LN(crown length)]				Wirth et al., 2004

*Total tree biomass is calculated as the sum of biomass of needles, branches, dry branches, the stem and roots.*

(4) $${\mathrm{sensitivity}}_{i}\;=\;\frac{2*{|increment}_{i}-{\mathrm{increment}}_{i}{{}_{-}}_{1}|}{\left({\mathrm{increment}}_{i}{+increment}_{i}{{}_{-}}_{1}\right)}$$

where |increment_i_ – increment_i-1_| is the absolute value of the difference between the increment of the current and preceding year.

For the biomass increment-climate correlation analysis we removed the decadal and longer scale trends from the biomass increment series in order to remove the influence of confounding effects such as age and competition dynamics from the data. The biomass increments were first power transformed and the optimal power (*p*) was computed as

(5) p = 1−m,

where m is the slope of the regression of the log10 median ring width against the log10 interquartile range of ring width based on non-overlapping, 10-year segments (Emerson, 1983). We obtained an optimal power of *p* = 0.14 (*N* = 3779, *r* = 0.82). We fit a 30-year cubic smoothing spline with a 50% frequency cutoff to each transformed tree biomass increment series and subtracted the spline from the transformed increments (Cook and Peters, 1997). The detrending procedure was performed using the package dplR (Bunn, 2008) in the R statistical software (version 3.1.1; R Development Core Team, 2014). This procedure removes not only the above-mentioned confounding effects, but also decadal scale climate-related trends (e.g., Wilson et al., 2005). To account for this trend removal, the climatic data were detrended following the same procedure.

Gridded monthly climatic data from the CRU TS3.10 database (Harris et al., 2014) were used for the biomass increment-climate correlation analysis. We selected two pairs of variables that could potentially limit photosynthesis or water availability [i.e., temperature, cloud cover, precipitation and Palmer drought severity index (PDSI)]. PDSI is a normalized index based on a water balance model of soil moisture where values around zero represent normal conditions and increasingly negative (positive) values represent progressively drier (wetter) conditions. We then performed a preliminary increment-climate correlation analysis (for the whole 1901 – 2007 period) to determine for which months each of the climatic variables show a significant relationship with the annual biomass increment. The months with strongest relationships for each variable were used to examine potential changes in the relationship between each climatic variable and biomass increment over time. If relevant the individualmonths with strong correlation were averaged (or summed for precipitation) into periods to obtain stronger relationship. Highest correlations were identified with growing season temperatures (May to September), cloud cover of the early growing season (May to July), precipitation in the late growing season of the previous year (previous-year July to previous-year September) and drought PDSI in September of the previous year, which is consistent with other studies of the same forest type (Levanič et al., 2009; Treml et al., 2012; Primicia et al., 2015). Mean correlations of average biomass increment series were 0.38 with temperature, 0.36 with cloud cover, 0.24 with precipitation and 0.19 with drought index (all relationships were statistically significant at *p* < 0.05 except drought index for the Čertovo catchment). Finally, we calculated the Spearman correlation coefficient between the detrended climatic variable series of the above-specified months and the detrended biomass increment series of each individual tree for the periods defined using temporal trend of air pollution (**Figure 1B**). The use of Spearman correlation was suitable here because of the relatively short periods used and the robustness of Spearman correlation against the effect of single extreme values. Nevertheless, the interpretation of increment-climate correlations should be viewed with caution considering the short periods examined. To support our interpretations we also present running Spearman correlations in the Appendix to acknowledge the temporal variability of the increment-climate relationship.

### Statistical Analysis

To assess the temporal trend of the physiological parameters in relation to air pollution, we averaged all of the parameters into periods defined by the air pollution trend (**Figure 1B**) so that for each period we obtained one value for each individual tree (see Supplementary Figures S1, S2 and S3 for the original time series). Using R software (version 3.1.1; R Development Core Team, 2014) for statistical analysis, we applied linear mixed effect models in the package lme4 (Bates et al., 2015) to test if the physiological parameters differed between the defined periods. The analysis was followed by Tukey’s pairwise comparison in the ‘lsmeans’ package (Lenth, 2015). We included tree individuals as a random effect and the time periods and catchment as a fixed effect. The catchment effect was significant in some models (see **Table 2**) and we present the effect in figures only for those cases where catchment effect was found to be significant. For Δ^13^C we also calculated a more complex model which included the biomass increment, inter-annual variability of the biomass increment, elevation, age, and diameter. Of these variables only the biomass increment showed a significant relationship. We selected the best model using the lowest Akaike Information Criterion (AIC) values and ANOVA comparison. We also calculated a pseudo-R^2^ based on Nakagawa and Schielzeth (2013) using the package piecewiseSEM (Lefcheck, 2015). Calculated pseudo-R^2^ comprises of marginal (R^2^m) and conditional (R^2^c) values that account for the proportion of the variance explained by the fixed factors and whole model (i.e., fixed plus random factors), respectively.

**Table 2:** Parameter estimates of the best mixed–effect models including tree–individual as a random effect.

	1971–1989	1998–2005	2006–2011	Plešné catchment	1971–1989 × Plešné catch.	1998–2005 × Plešné catch.	Intercept	R^2^m^1^	R^2^c^1^	AIC
Δ^13^C^2^	-1,876	-0,696	-0,480				19,635	0,52	0,87	143
Ci^2^	-4,060	35,217	44,241				204,310	0,80	0,94	672
iWUE^2^	24,604	21,056	20,807				61,944	0,58	0,89	578
Biomass increment	1,141	2,468		3,702	–1,966	0,083	9,945	0,05	0,71	4157
Inter-annual variability^3^	0,090	0,041					0,170	0,22	0,51	–1648
Temperature correlation^4^	0,236	0,167		0,224	–0,159	–0,080	-0,018	0,21	0,29	–221
Precipitations correlation^4^	-0,024	0,039		0,065	0,029	0,091	0,111	0,12	0,12	–279
Cloud cover correlation^4^	-0,214	-0,183		–0,067			-0,039	0,18	0,18	–140
Drought index correlation^4^	-0,009	0,137		0,013	0,039	0,197	0,029	0,26	0,27	–189

	**1971–1989**	**1998–2005**		**Δ^13^C**	**1971–1989 × Δ^13^C**	**1998-2005 × Δ^13^C**	**Intercept**			

Biomass increment	-108,637	12,427		-5,285	5,473	-0,871	120,677	0,09	0,84	290

*The temporal trend was assessed in periods defined based on the temporal pattern of atmospheric pollution (**Figure 1B**), i.e., before (1900–1935), during (1971–1989) and after (1998–2005 and 2006–2011) the peak in air pollution. The model shows parameter estimates relative to the 1900–1935 period and Čertovo catchment. Only significant effects are shown.*

*^1^Marginal (proportion of variance explained by the fixed factor, R^2^m) and conditional (proportion of variance explained by fixed plus random factors, R^2^c) pseudo-R^2^ values were calculated following Nakagawa and Schielzeth (2013).*

*^2^Δ^13^C, Ci and iWUE represent carbon isotope discrimination, intercellular CO_2_ concentration and intrinsic water use efficiency.*

*^3^Inter-annual variability of biomass increment calculated as the dendrochronological metric called sensitivity (Fritts, 1976).*

*^4^Spearman correlations of detrended biomass increment-climate relationship.*

## Results

**FIGURE 2:**
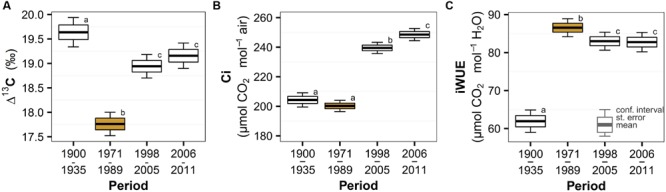
**Temporal trend of Norway spruce **(A)** carbon isotope discrimination (Δ^13^C), **(B)** intercellular CO_2_ concentration (Ci) and **(C)** intrinsic water-use efficiency (iWUE) divided into periods based on the trend of atmospheric pollution (the period with peaking pollution is orange highlighted) in the Bohemian Forest.** Significant changes are represented by different lowercase letters.

We found significant temporal changes in spruce physiological parameters in response to the peak in air pollution (**Table 2**), but the temporal trend of the individual parameters was different. The most distinct deviation during the air pollution period was observed in the discrimination against heavier carbon isotope (Δ^13^C, **Figure 2A**) that transiently decreased and recovered after the pollution period. This result indicates a relatively lower internal leaf CO_2_ concentration (in comparison to atmospheric CO_2_) and most likely stressful conditions due to polluted air. Model estimates (**Table 2**) show a 10% decrease in Δ^13^C in response to the pollution and a subsequent recovery of 7%. Surprisingly, considering the atmospheric CO_2_ concentration increase (**Figure 1B**), it seems that intercellular CO_2_ availability remained relatively stable during the peak in pollution compared to the earlier period (**Figure 2B**). We observed a slight, though insignificant, decrease of Ci by 2% in the pollution period, but a sharp increase in the recent periods by 17 – 24% likely as a result of release from polluted conditions and higher CO_2_ availability. The combined effect of air pollution and CO_2_ resulted in changes of spruce water use efficiency (**Figure 2C**), which sharply increased (by 40%) during the peak in air pollution and decreased slightly (by 4%) after pollution levels declined. Water use efficiency was highest during the pollution period, but the effect of increased atmospheric CO_2_ can be observed in the difference between the iWUE before and after the pollution (34% increase).

**FIGURE 3:**
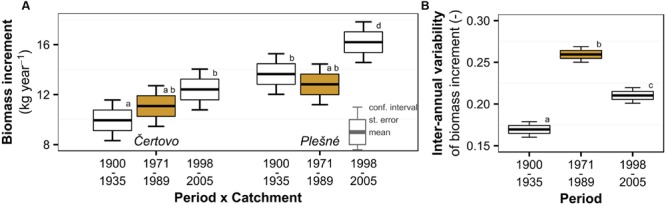
**Temporal trend of Norway spruce **(A)** biomass increment and **(B)** its inter-annual variability divided into periods based on the trend of atmospheric pollution (the period with peaking pollution is orange highlighted) in the Bohemian Forest.** Significant changes are represented by different lowercase letters.

Mean tree biomass increment did not show any consistent temporal trend with air pollution or Δ^13^C since the periods before and during pollution did not differ significantly (**Figure 3A**). However, we observed a slight increase in the recent period after the pollution diminished, though significant at Plešné (26% increase) and insignificant at Čertovo (12% increase) catchment. On the other hand, inter-annual variability of the biomass increment followed a similar trend to Δ^13^C and air pollution (**Figure 3B**), which may suggest increased sensitivity to environmental factors induced by pollution. We observed a 53% increase in inter-annual variability during the pollution period relative to the pre-pollution period and a subsequent decrease of 19% after pollution levels decreased. The biomass increment generally reflects diverse environmental factors (such as age or competition) as displayed in the lowest explained variance in the models of biomass increment and the involvement of catchments ant catchment–period interactions.

**FIGURE 4:**
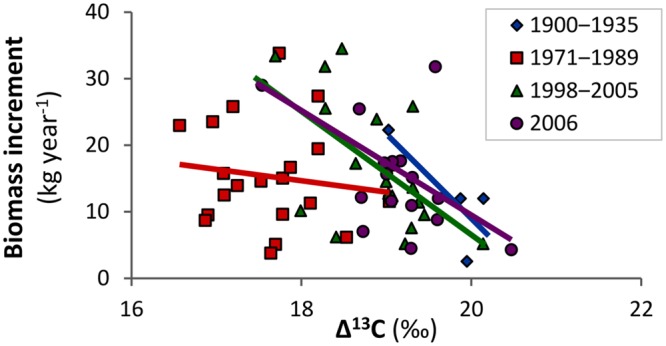
**Biomass increment of mountain Norway spruce is significantly related to carbon isotope discrimination (Δ^13^C) prior to and after the period affected by air pollution, but the relationship was lost during the peak in air pollution (1971–1989) in the Bohemian Forest**.

At the individual tree level, biomass increment was significantly related to variables obtained from isotopic analysis. **Figure 4** shows the relationship of individual tree biomass increment with Δ^13^C and indicates that the growth of trees which fixed more of the heavier isotope was more vigorous. The relationship was similar for periods before and after the peak in air pollution, but was non-significant for the air pollution period (as the model included interactions; **Table 2**). This result suggests that the increased fixation of ^13^C during air pollution was more prominent in trees, which grew slowly, and the influence of air pollution on Δ^13^C likely overrode other environmental effects (expressed in the biomass increment).

**FIGURE 5:**
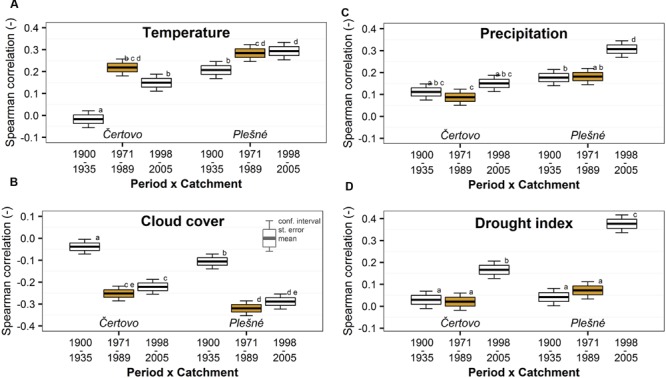
**Temporal trend of Spearman correlations between biomass increment and growing season **(A)** temperatures (May–September), **(B)** cloud cover in the early growing season (May–July), **(C)** precipitation in the previous late growing season (previous July–September) and **(D)** drought (Palmer drought severity index) of the previous September.** The trend was divided into periods based on the trend of atmospheric pollution (the period with peaking pollution is orange highlighted) in the Bohemian Forest. Significant changes are represented by different lowercase letters.

We found no clear trend consistent with air pollution when examining the increment-climate relationships (**Figure 5** and Supplementary Figure S3). Correlations with variables potentially limiting photosynthesis (i.e., temperatures and cloud cover) were weaker at the beginning of the 20th century but remained stable in later periods. Correlations between biomass increment and variables potentially indicating water stress (i.e., precipitation and drought) increased in the last few years, indicating that severe drought has an effect on the increment. However, we observed no significant effect of air pollution. Catchment effect and catchment–period interactions were again significant and Plešné catchment trees showed generally better climate relationships. Older ages or lower competition pressure at Plešné catchment are the possible explanations for this pattern.

## Discussion

We investigated the extent to which moderate air pollution affected the physiology of Norway spruce. We found that the physiology was significantly influenced, but different physiological parameters showed variable responses because of the interaction with increasing atmospheric CO_2_ concentrations and increasing temperatures due to climate change. As a result, the biomass increment did not change significantly during the peak in air pollution, yet increased slightly in the recent period. Biomass increment was inversely related to carbon isotope discrimination at the individual tree level, but the relationship was lost during the pollution period. As our results did not indicate increased water stress or decreased influence of climate on photosynthesis in response to the pollution (because tree biomass increment did not become more or less sensitive to drought/precipitation or temperature/cloud cover, respectively), we suggest that the direct influence of pollution on stomatal conductance was the main driver of the observed physiological changes (see below).

Carbon isotope discrimination is a sensitive indicator of the physiological response of plants to air pollution (particularly SO_2_ deposition; Savard, 2010). We found a decrease of 1.88‰ Δ^13^C in response to the deposition of, particularly, 124 mmol SO_4_^2-^ m^-2^ year^-1^ and 95 mmol NO_3_^-^ m^-2^ year^-1^. This Δ^13^C decrease is in the middle of the range of values reported in other studies, where the decrease ranged from 1‰ to more than 3‰ in both conifers and deciduous species (Niemelä et al., 1997; Rinne et al., 2010; Savard, 2010; Thomas et al., 2013). The Δ^13^C decrease exceeds the typical range of natural variation (usually less than 0.5‰) and is proportional to the pollution load (Niemelä et al., 1997; Savard, 2010), which was at moderate level in our study area. Our results demonstrate that changes in Δ^13^C can be used as a sensitive indicator of acid pollution stress far earlier than tree mortality, reduction of growth or possibly even before reduction in the rate of photosynthesis occurs (Thomas et al., 2013). We also provide evidence that air pollution significantly affected tree physiology throughout central Europe and not only in the most polluted regions such as the “Black Triangle” as suggested by Kandler and Innes (1995).

The effects of air pollution on tree physiology interacted with other environmental changes such as the increase in atmospheric CO_2_ or temperature. Observed 20th century increases of iWUE in many ecosystems can be related not only to the increase in atmospheric CO_2_ (Peñuelas et al., 2011; Saurer et al., 2014), but also to increased atmospheric pollution. The iWUE significantly increased by 3.8 μmol CO_2_ mol^-1^ H_2_O in the polluted conditions with 339 ppm CO_2_ in comparison to the recent post-pollution conditions when the concentration of atmospheric CO_2_ reached 381 ppm. Increased atmospheric CO_2_ can compensate for the negative effects of air pollution because the Ci can remain similar (Thomas et al., 2013). Despite the major iWUE increase in the second half of the 20th century in our study location, the biomass increment did not increase correspondingly, suggesting that there is no causal relationship between the two variables and that an increase in iWUE does not necessarily mean increase in the carbon storage of the forest (Peñuelas et al., 2011).

Compensation of the negative effect of pollution by increased ambient CO_2_ concentrations which produced comparable Ci before and during the pollution period could partly explain why the biomass increment did not change significantly. Similarly to CO_2_, air temperature, which is a limiting factor for spruce biomass increment in our study area, increased during the 20th century and could have therefore also mitigated the negative effect of air pollution (Levanič et al., 2009; Treml et al., 2012; Primicia et al., 2015). It is also possible that the carbon partitioning pattern of the trees may have changed in favor of above-ground biomass during the pollution period resulting in unchanged biomass increment derived from stem diameter increments. The negative effects of high nitrogen load on trees (Högberg et al., 2006) and particularly on below-ground carbon allocation (root growth and mycorrhiza; Jentschke et al., 2001; Nilsson and Wallander, 2003) have been documented. Similarly, rather than affecting biomass increment in terms of volume, air pollution could affect wood density (Sander et al., 1995). Generally, the stem volume increment is probably affected when air pollution exceeds some threshold value, as occurred in the most polluted part of central Europe (Hauck et al., 2012; Rydval and Wilson, 2012).

However, the sensitivity of different tree species to pollution is likely variable. Silver fir (*A. alba*) was found to be highly sensitive with growth significantly affected even in the moderately polluted region of southern Germany near our study area (Elling et al., 2009). In addition to the effect on the growth increment, the fir carbon isotope ratio changed by about 3‰ in this area and the oxygen isotope ratio also showed a distinct effect of pollution on fir physiology (Boettger et al., 2014). The physiology of other coniferous (e.g., pine *Pinus sylvestris*) and deciduous (e.g., oak *Quercus robur*) species was also found to be influenced by pollution (Rinne et al., 2010). *Pinus sylvestris* was observed to react sensitively even to low pollution loads (Niemelä et al., 1997). However, direct comparisons of the response of different tree species to pollution, excluding the influence of site, climatic and environmental conditions and variability in pollutant deposition, are required, particularly because these factors significantly influence the specific reaction of each species to the pollution load. For instance, mountain areas with shallow and acidic soils are the most sensitive to acid pollution and conifers, which have a relatively large leaf area that remains exposed during the winter, are subjected to higher total amounts of pollutant – a factor unrelated to their physiological resistance (Kopáček and Hruška, 2010; Greaver et al., 2012).

The biomass increment increase in the recent period may not be related only to the direct effects discussed above, but also to the complex ecosystem release from the acidic conditions. These are related for example to increased decomposition of organic matter accumulated during the pollution period, its utilization (cessation of nitrate leaching) and improved nutrition as indicated by needle nitrogen concentrations (Oulehle et al., 2011). Changes in between tree competitions related to disturbance events could also play a role in determining changes in biomass increment and particularly in the higher recent growth increase at Plešné catchment, which was affected by bark beetle outbreak since 1990s (Svoboda et al., 2012; Janda et al., 2014). As our results indicate that extreme droughts periodically influenced the increment, an increase in the frequency of drought years due to climate change can also lead to the increasing importance of drought as a stress factor that negatively influences spruce biomass increment (Primicia et al., 2015).

Inter-annual variability of the biomass increment increased significantly during the pollution period. This may be related to the increased sensitivity of pollution affected trees to other stress factors such as frost, insects or drought (Kandler and Innes, 1995). Moderate pollution loads probably do not directly cause widespread tree damage as observed in most polluted regions, but increase tree vulnerability to other stressors, which can in combination cause forest decline (Kandler and Innes, 1995). Increased variability is thought to be an indicator of increased stress or an “early warning signal” for a critical state transition (Scheffer et al., 2009). At the same time we cannot discount the possibility that this result was related to a series of favorable and unfavorable growth years which may have occurred during the air pollution period.

Air pollution can influence trees via several pathways, i.e., directly through foliage or indirectly through the soil. Soil acidification decreases soil nutrient availability and often mobilizes aluminum, which is toxic to plants and can obstruct nutrient uptake (Jentschke et al., 2001; Hruška et al., 2012). Our biomass increment-climate correlation analysis does not support the hypothesis that possible root weakness caused water stress in our study site. Nutrient deficiency can lead to a reduction in photosynthesis rate. While decreased capacity of photosynthesis alone would lead to an increase in Δ^13^C (followed by increased Ci), we instead observed a decrease in Δ^13^C and therefore refute the notion that soil acidification would be the main driver of the observed physiological changes (Viet et al., 2013).

A direct effect of pollution on foliage can decrease the rate of photosynthesis and/or stomatal conductance (Meng et al., 1994). Our results, which show a reduction of Δ^13^C during the air pollution period, do not support the pollution influence via reduced photosynthesis alone, but are instead in line with decreased stomatal conductance (Farquhar et al., 1982; McCarroll and Loader, 2004). No consistent trend in the correlation between biomass increment and temperature or cloud cover, which would have also indicated photosynthesis limitation, supports the idea that stomatal conductance was the main driving force of the observed Δ^13^C decrease. Minor limitation of photosynthesis in response to either the direct or indirect pollution impact could be indicated by physiological patterns such as non-significantly decreased biomass increment and Ci or greatest iWUE during pollution period. Increased dark respiration was also proposed to be related to air polluted conditions and could have also played a role (Savard, 2010). We suppose that photosynthesis limitation and root weakening are of higher importance in locations with different site conditions or a higher pollution load as suggested by some studies showing decreased responses to temperature (Sander et al., 1995; Elling et al., 2009) or in more sensitive species (Boettger et al., 2014). Our interpretation is also supported by Kandler and Innes (1995) that reviewed in situ measurements of gas exchange done around the year 1990 and found no influence on photosynthesis rate in moderately polluted areas, but significant effect in most polluted areas. We therefore conclude that moderate pollution levels have the greatest effect directly on stomatal conductance (Thomas et al., 2013), which increases tree stress and sensitivity to other stressors.

The negative relationship between Δ^13^C and biomass increment was significant in the periods before and after the pollution, while it was lost during the pollution period. The positive or negative relationship between Δ^13^C and growth was used to indicate stomatal or non-stomatal (i.e., carboxylation rate) limitation of assimilation in trees (Viet et al., 2013). The relationship was positive in *P. strobus* limited by soil water availability and, presumably, stomatal conductance (McNulty and Swank, 1995), while it was negative in *P. densiflora*, where photosynthesis was limited by soil acidity. In our case, air temperature is the main climatic variable driving physiological processes of mountain Norway spruce growing in non-polluted conditions (Levanič et al., 2009; Treml et al., 2012; Primicia et al., 2015) and the negative relationship between Δ^13^C and biomass increment supports the statement about the dominant control of the rate of photosynthesis on Δ^13^C in non-polluted conditions. This implies that trees exposed to more sunlight grow better, utilize intercellular CO_2_ faster and their Δ^13^C is therefore lower in comparison to more slowly growing shaded trees. We therefore suggest that the loss of the negative relationship between biomass increment and Δ^13^C during the pollution period in our study area indicates a loss of photosynthetic rate as the dominant control of Δ^13^C in favor of stomatal conductance. This supports our conclusion that moderate pollution predominantly influenced studied physiological processes by reducing stomatal conductance.

## Conclusion

Moderate levels of atmospheric pollution and particularly sulfate and reactive nitrogen acid deposition significantly affected the physiology of mountain Norway spruce in central Europe. We observed a complex response of spruce trees to pollution with Δ^13^C being the most sensitive indicator of the pollution effect. The atmospheric pollution effects interacted with those of increasing atmospheric CO_2_ and temperature resulting in unchanged intercellular CO_2_ concentrations abruptly increased water use efficiency and unchanged biomass increment, which could also partly result from changes in carbon partitioning. Our biomass increment-climate correlation analyses did not indicate any clear pollution-related changes in water stress or photosynthetic limitation due to climate. We therefore concluded that the direct effect of moderate pollution on stomatal conductance was likely the main driver of the observed physiological changes. We also assume that the shift from the dominant control of the photosynthetic rate to the stomatal conductance on Δ^13^C during the pollution period caused the loss of the negative relationship between biomass increment and Δ^13^C. This mechanism probably caused weakening of the spruce trees and increased sensitivity to other stressors, as indicated by increased inter-annual variability of biomass increment.

## Author Contributions

HŠ, LK and MSv designed the research and data collection. JŠ and LK performed the laboratory analysis. VČ, HŠ, LK and MSe did the calculations and statistical analysis. VČ organized the manuscript preparation with the contribution of all the authors.

## Conflict of Interest Statement

The authors declare that the research was conducted in the absence of any commercial or financial relationships that could be construed as a potential conflict of interest.

## Footnotes

1 http://www.emep.int

2 http://www.rinntech.com
