# Supplementary material for: Complex Physiological Response of Norway Spruce to Atmospheric Pollution – Decreased Carbon Isotope Discrimination and Unchanged Tree Biomass Increment
Source: Front Plant Sci. 2016 Jun 9;7:805. doi: 10.3389/fpls.2016.00805 (PMC4899467; doi:10.3389/fpls.2016.00805)
Supplement: Supplementary file 1 [file Data_Sheet_1.PDF]

Supplementary Material

Complex physiological response of Norway spruce to atmospheric pollution – decreased carbon isotope discrimination and unchanged tree biomass increment

Vojtěch Čada*, Hana Šantrůčková, Jiří Šantrůček, Lenka Kubištová, Meelis Seedre, Miroslav Svoboda

*** Correspondence:** Vojtěch Čada: cada@fld.czu.cz

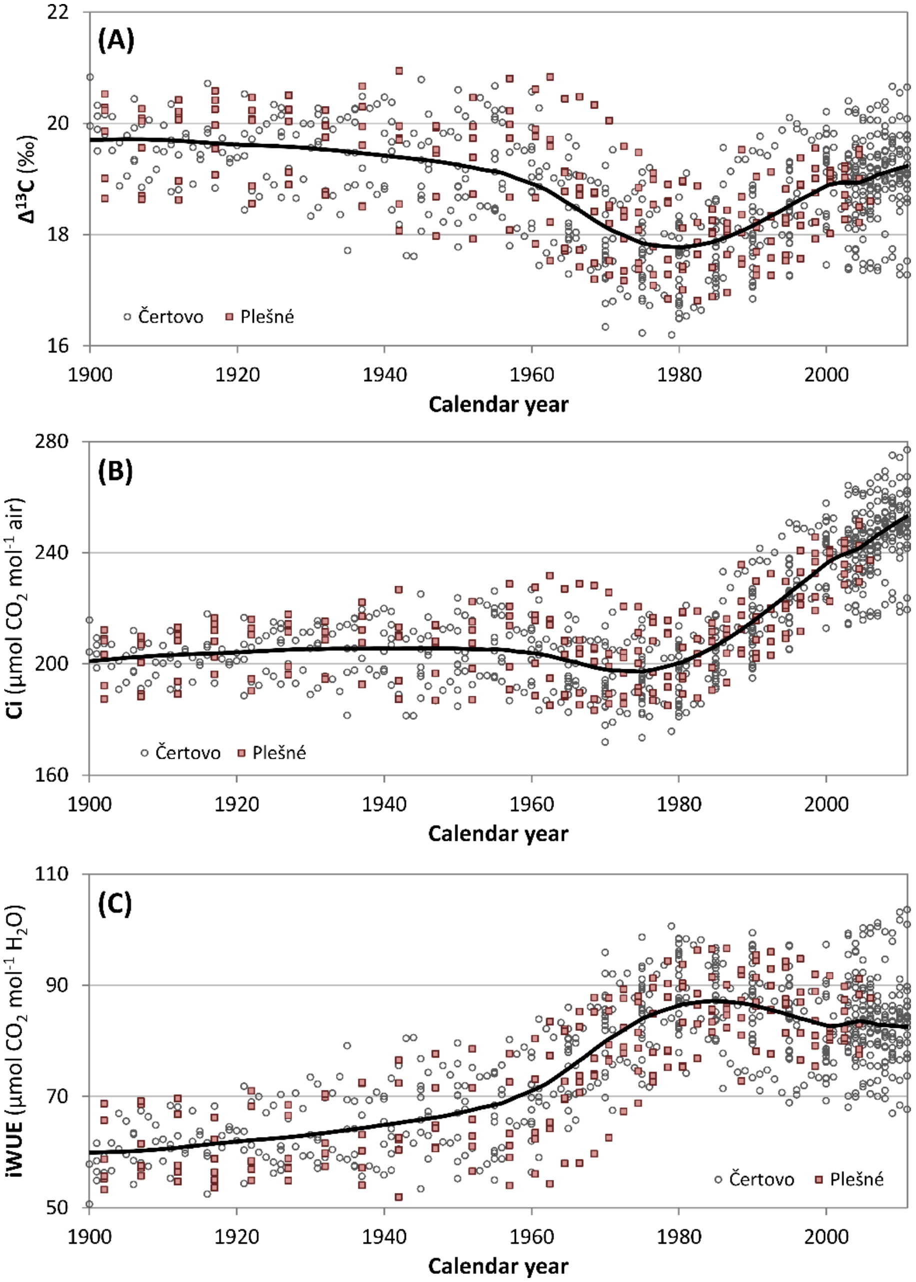

Supplementary Figure S1. Time series of **(A)** carbon isotope discrimination ($\Delta^{13}C$), **(B)** intercellular CO_2 concentration (Ci) and **(C)** intrinsic water-use efficiency (iWUE) of Norway spruce trees in two locations of the Bohemian Forest including lowess fit.

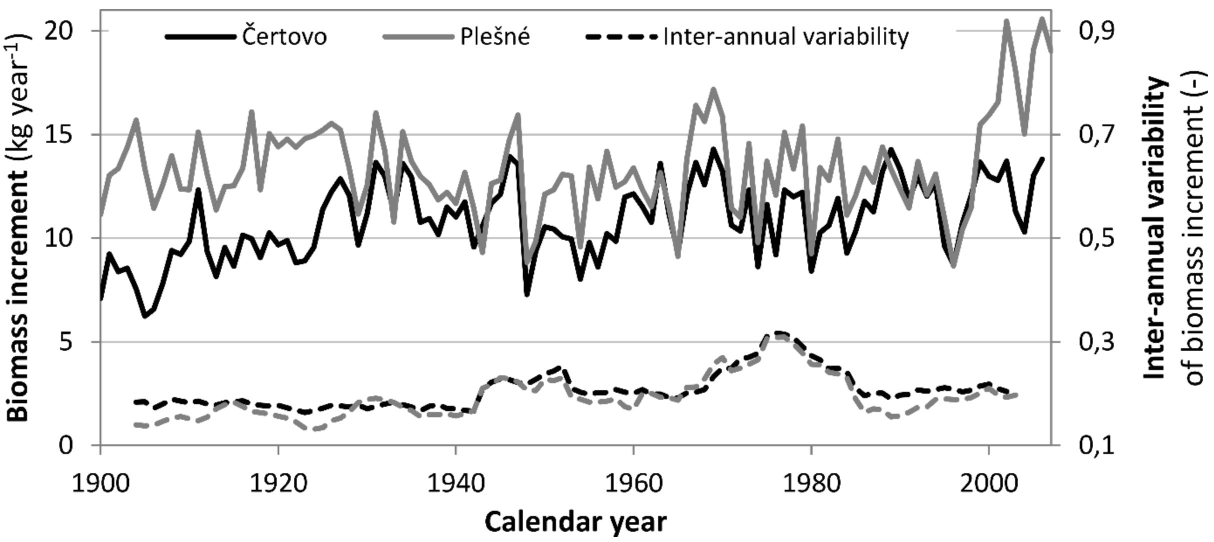

Supplementary Figure S2. Time series of average biomass increment and 9-year running average of biomass increment inter-annual variability for Norway spruce in two locations in the Bohemian Forest.

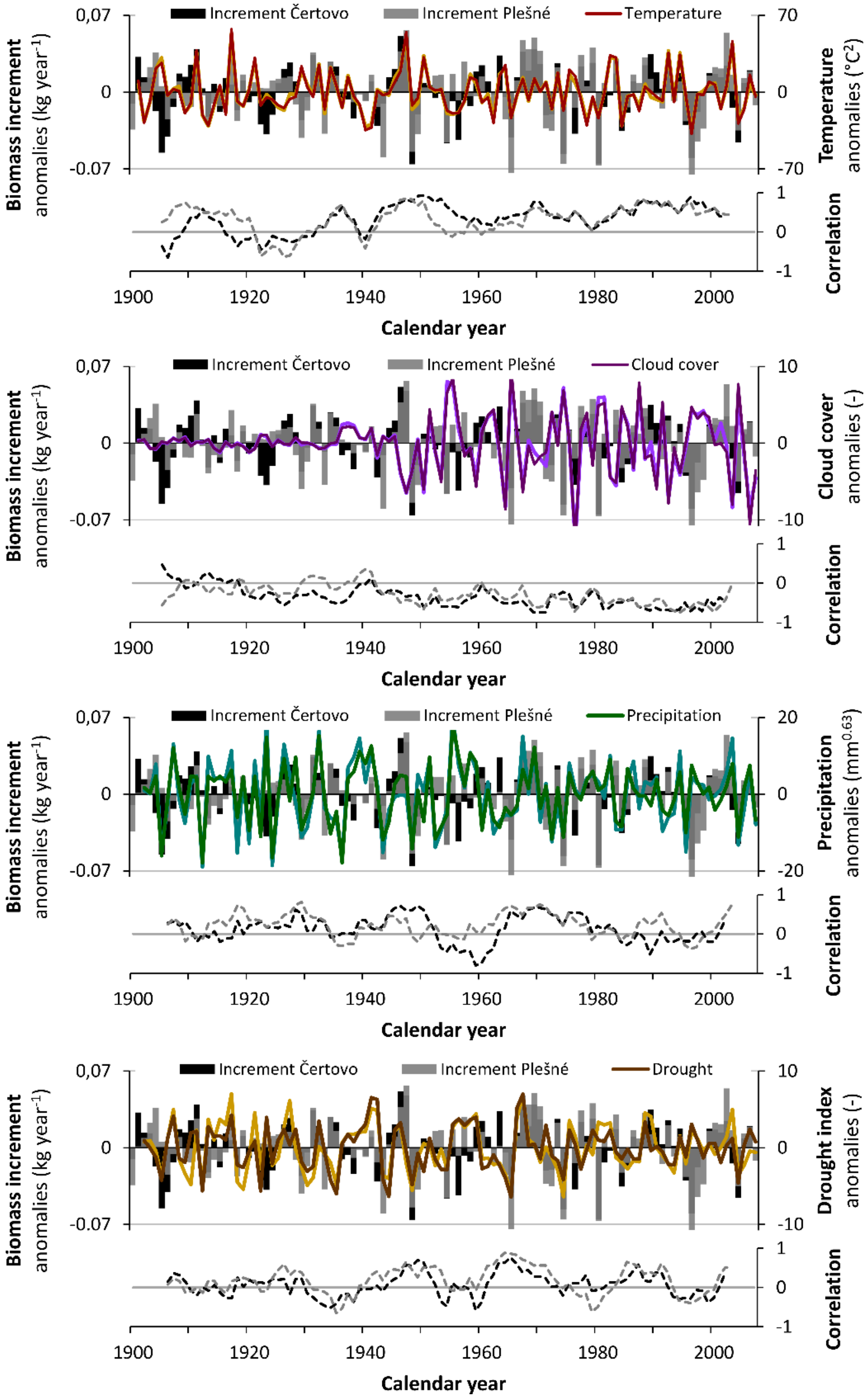

Supplementary Figure S3. Time series comparison of average detrended Norway spruce biomass increment series (columns) with detrended climatic data (lines) for the Čertovo (dark colors) and Plešné (light colors) locations in the Bohemian Forest. Temporal changes in association between increment and climatic variables are expressed with a 9-year running Spearman correlation below the time series.
